# A Novel Function for Kojic Acid, a Secondary Metabolite from *Aspergillus* Fungi, as Antileishmanial Agent

**DOI:** 10.1371/journal.pone.0091259

**Published:** 2014-03-12

**Authors:** Ana Paula D. Rodrigues, Luis Henrique S. Farias, Antonio Sérgio C. Carvalho, Alberdan S. Santos, José Luiz M. do Nascimento, Edilene O. Silva

**Affiliations:** 1 Universidade Federal do Pará, Instituto de Ciências Biológicas, Laboratório de Biologia Estrutural, Belém, Pará, Brazil; 2 Universidade Federal do Pará, Instituto de Ciências Exatas e Naturais, Laboratório de Investigação Sistemática em Biotecnologia e Biodiversidade Molecular do Instituto de Ciências Exatas e Naturais, Belém, Pará, Brazil; 3 Universidade Federal do Pará, Instituto de Ciências Biológicas, Laboratório de Neuroquímica Molecular e Celular, Belém, Pará, Brazil; 4 Instituto Nacional de Ciência e Tecnologia em Biologia Estrutural e Bioimagem, Universidade Federal do Rio de Janeiro, Rio de Janeiro, Rio de Janeiro, Brazil; 5 Laboratório de Microscopia Eletrônica, Instituto Evandro Chagas, Secretaria de Vigilância em Saúde do Ministério da Saúde, Belém, Pará, Brazil; University of California Merced, United States of America

## Abstract

Kojic acid (KA) is a fungal metabolite used as a topical treatment skin-whitening cosmetic agent for melasma in humans; however its potential as an anti-leishmanial agent is unknown. Chemotherapy is one of the most effective treatments for Leishmaniasis. However, the drugs available are expensive, invasive, require long-term treatment and have severe side effects. Thus, the development of new effective leishmanicidal agents is a necessity. In this study we investigated the anti-leishmanial effect of KA on *L. amazonensis*, following *in vitro* and *in vivo* infections. KA (50 μg/mL) was found to decrease the growth by 62% (IC_50_ 34 μg/mL) and 79% (IC_50_ 27.84 μg/mL) of promastigotes and amastigotes *in vitro*, respectively. Ultrastructural analysis of KA-treated amastigotes showed the presence of vesicles bodies into the flagellar pocket, and an intense intracellular vacuolization and swelling of the mitochondrion. During the *in vitro* interaction of parasites and the host cell, KA reverses the superoxide anions (O_2_^-^) inhibitory mechanism promoted by parasite. In addition, 4 weeks after KA-topical formulation treatment of infected animals, a healing process was observed with a high production of collagen fibers and a decrease in parasite burden. Thus, these results demonstrated the great potential of KA as an anti-leishmanial compound.

## Introduction

Leishmaniasis is a disease caused by protozoan parasites of the genus *Leishmania*. Parasites are transmitted by sandflies and can infect cells of the mononuclear phagocyte lineage in vertebrate hosts. Leishmaniasis is endemic in 98 countries and affects 12 million people in the entire world available in the WHO homepage (http://www.who.int/tdr/diseases/leish/diseaseinfo.htm). The disease is a public health problem in Brazil, particularly in the Amazon region, due to the presence of seven different enzootic species of *Leishmania*, involving hosts and different sand fly vectors that are commonly found in this region [1]. Leishmaniasis severity varies, extending from mucosal and cutaneous to visceral and diffuse cutaneous infections [2]. Diffuse cutaneous leishmaniasis or anergic diffuse cutaneous leishmaniasis (ADCL) is caused by *Leishmania (Leishmania) amazonensis*, which causes cellular immune response depression, results in parasite-rich lesions and is characterized by non-ulcerated lesions.

Chemotherapy is one of the most effective treatments for this disease. The first line of treatment recommended by the WHO consists of the use of pentavalent antimonials, amphotericin B and pentamidines, which have demonstrated treatment failure and parasite resistance. In addition, these treatments are expensive, invasive and have severe side effects. Treatment for ADCL is not effective for some patients due to the anergic response profile [3]. Alternative treatments are available, such as miltefosine, which is effective against visceral leishmaniasis in India, but is teratogenic and few studies have shown effects on tegumentary leishmaniasis [4]. New substances, isolated from plants and microorganisms, have demonstrated leishmanicidal action and most act by promoting host cell activation to combat leishmania parasites [5], [6].

5-Hydroxy-2-hydroxymethyl-γ-pyrone (HMP) or kojic acid (KA), produced by some species of *Aspergillus* fungi, is a water-soluble secondary metabolite. KA is used as a food additive [7], [8], [9], as a skin-whitening cosmetic agent for the treatment of melasma [10], [11], [12], [13], an antioxidant, antitumor agent [7], [14], [15], [16] and radioprotective agent [17]. Recently, we have shown that KA is able to activate mice peritoneal macrophages, promoting O_2_^-^ production, enhanced phagocytosis activity and cytoskeleton reorganization [18]. It should be empathized that, based on previous and current data obtained by our group, three patent applications have been registered and published [19], [20], [21] proposing the use of KA as an anti-leishmanial product. Furthermore, only one study shows the action of KA on parasites, where it is reported to act by inhibiting a tyrosinase enzyme in *Schistosoma mansoni* [22]. However, no information is available regarding its effect on the *Leishmania* parasite. Thus, studying the effects of KA against *Leishmania (L.) amazonensis* parasites *in vitro*, is of interest; furthermore, this study also aimed to test the use of KA-topical formulation on experimental cutaneous leishmaniasis

## Materials and Methods

### Ethics statement

The study was carried out in strict accordance with the Brazilian animal protection law (Lei Arouca number 11.794/08) of the National Council for the Control of Animal Experimentation (CONCEA, Brazil). The protocol was approved by the Committee on the Ethics of Animal Experiments of the Federal University of Pará (CEPAE/ICB/UFPA - grant number BIO001-09).

### Animals

Eight-week-old female Golden hamsters (*Mesocricetus auratus*) and female BALB/c mice (6 to 8 weeks old) were obtained from the Evandro Chagas's Institute (Belém, Pará).

### Parasites

*Leishmania (Leishmania) amazonensis* (IFLA/67/BR/PH8) promastigotes were obtained from the Evandro Chagas's Institute and cultured at 26°C in NNN medium. Subsequently, promastigotes were cultured in RPMI medium supplemented with 10% fetal calf serum (FCS), 0.2 M glutamine, 0.125 M pyruvic acid and 5 mM penicillin/streptomycin.

### Kojic acid (KA) production

The secondary metabolite, 5-hydroxy-2-(hydroxymethyl)-γ-pyrone (HMP) or kojic acid (KA), is a molecule that is highly soluble in water, ethanol and acetone. KA was obtained by a biotechnological process, according to Ferreira *et al*. (2010) [23] and Rodrigues *et al*. (2011) [18].

### KA-topical formulation production and composition

Quantities of 100 mg of KA were prepared with tryacylglycerol from *Theobroma glandiflorum* seeds (FTGS) at a temperature of 30°C. The humectant agent (FTGS) was heated for 5 minutes and KA was added, followed by homogenization. FTGS was used as a carrier of KA to facilitate the penetration through the cell membrane.

### Murine macrophages

Resident macrophages were obtained from peritoneal cavities of BALB/c mice with Dulbecco's Modified Eagle's Medium (DMEM), pH 7.2, and incubated at 37°C in a humidified atmosphere containing 5% CO_2_. After 1 h of incubation, non-adherent cells were washed away with phosphate buffered saline (PBS), pH 7.2, and macrophages were incubated overnight in DMEM medium supplemented with 10% heat-inactivated fetal bovine serum (FBS) at 37°C and in a 5% CO_2_ atmosphere. All experiments were performed at least three times with treated and untreated cells.

### *In vitro* assays

#### a. Antipromastigote assay

*L. (L.) amazonensis* promastigotes (10^6^ parasites/mL) were inoculated in a 24-well plate containing RPMI medium supplemented with 10% inactivated fetal bovine serum treated with different concentrations of KA and incubated at 25 °C for 5 days without medium replacement. Every 24 h after treatment, aliquots were harvested and the effect of KA on promastigotes growth was evaluated using a Neubauer chamber and compared with untreated parasites culture. The cultures were performed in triplicate. Glucantime® was used as a positive control. The inhibitory concentration (IC_50_) was determined using SigmaPlot (version 12).

#### b. Intracellular amastigote assay

Adhered peritoneal macrophages were infected with *L. amazonensis* promastigotes (stationary growth phase) at a parasite/macrophage ratio of 10∶1 and incubated for 3 h at 37°C and 5% CO_2_. Subsequently, free parasites were removed by washing with phosphate-buffered saline (PBS) and cultures were treated with 10, 20 and 50 μg/mL of KA, for 1 h daily for 3 days post infection, replacing the culture medium every day. The treatment design (1 hour/day) showed good efficacy and was chosen based in previous data from our group^18^. Cells were then washed with saline solution, fixed in methanol and stained with Giemsa. The number of parasites was determined by examining three coverslips for each treatment. At least 200 infected macrophages were counted and results were expressed as infectivity index (infectivity index (II)  =  parasite internalized/cell × percentage of infected macrophages divided by the total number of macrophages). Glucantime® (50 μg/mL) was used as a positive control. The inhibitory concentration (IC_50_) was determined using SigmaPlot (version 12). Macrophages were treated with 20–1000 μg/mL using the same design applied for antiamastigote test describe above and MTT assay ([3-(4,5-dimethylthiazol-2-yl)-2,5-diphenyl tetrazolium bromide) was used to determine cell viability.

#### c. Transmission electron microscopy (TEM) of intracellular amastigotes

*L. amazonensis*-infected mouse peritoneal macrophages treated with 50 μg/mL of KA for 1 h and maintained for 24 h in culture were washed in PBS and fixed with 2.5% glutaraldehyde, 4% formaldehyde in 0.1 M sodium cacodylate buffer, pH 7.2, and post-fixed in 1% osmium tetroxide, 0.8% ferrocyanide, dehydrated in graded acetone and embedded in epoxy resin. Ultrathin sections obtained were stained with uranyl acetate and lead citrate and examined with a Zeiss 906E TEM.

#### d. Superoxide anions detection and nitric oxide production in infected macrophages treated with KA

The detection of superoxide anions (O_2_^−^) was performed using cytochemical detection with nitroblue tetrazolium salt (NBT). NBT is a yellow dye that is converted to blue by a semiquantitative reduction reaction when superoxide anion is present in cells. The experiments were performed according to Rodrigues *et al*. (2011) [18] with some modification. After 24 h of growth, macrophages were incubated with 50 μg/mL of KA, 0.5 mg/mL of NBT and in the presence or absence of *L.amazonensis* promastigotes (ratio 10∶1) for one hour. For each slide, approximately 100 infected macrophages were examined and counted. Cells were differentiated as infected macrophages that presented O_2_^−^ reaction and macrophages infected that not presented O_2_^−^ reaction (SO^−^). Results were presented as number of infected cells showed formazan deposits. The supernadant of macrophages infected and treated were used for nitrite detection by griess reaction.

### *In vivo* assays

#### a. Antileishmanial experiment

Animals (eight-week-old female Golden hamsters) were infected with 10^6^ of *L. amazonensis* promastigotes/mL during the stationary growth phase with a maximum volume of 0.2 mL on both hind paws. Animals were separated in 3 groups: untreated (n = 5); KA-treated 100 mg/kg/day (n = 5) and KA vehicle (n = 5). KA topical formulation treatment was initiated after 5 weeks of infection. The KA formulation and vehicle was applied topically to all lesions once daily for 4 weeks. Control groups were also maintained in parallel. During the treatment period, the lesion size was measured weekly using a caliper. Width and height of both hind paws were used to calculate the lesion area (mm^2^). After the treatment, animals were euthanized and tissues from lesions were processed for histopathological analysis, scanning and transmission electron microscopy analysis.

#### b. Histophatological analysis

Tissue biopsies from infected and treated animals were fixed and embedded in paraffin. Sections of 5–6 mm were stained with haematoxilin and eosin (H&E) for histopathological analysis. Morphometric analysis was employed to quantify tissue parasitism as described by Rocha-Vieira *et al*. (2003) [24]. Images from tissues were captured with a Zeiss Axiophot microscope connected to a video camera. Quantitative morphometric analysis was performed using ImageJ software and digitalized images obtained. The parasite number was measured in 10 non-contiguous fields from both infected footpads of each animal (magnification 630 x).

#### c. Transmission and scanning electron microscopy

Tissue sections from infected and treated animals were processed as described above for TEM and examined with a LEO 906E TEM. For SEM, tissue was processed according to Haggis *et al*. (1977) [25]. Samples were fixed and post-fixed as described above for TEM, dehydrated in graded ethanol, frozen using liquid nitrogen, fractured and, after thawing, critical-point dried. Samples were mounted, coated with gold and examined with a LEO 1450VP SEM.

### Statistical analysis

All experiments were performed in triplicate. The mean and standard deviations of at least three experiments were determined. Statistical analyses of the differences between mean values in the experimental groups were performed using ANOVA, the Student's t-test (employing the GraphPad Prism 5.0 program). All p-values <0.05 were as considered statistically significant.

## Results

### KA has antileishmanial activity, *in vitro*

**Figure 1:**
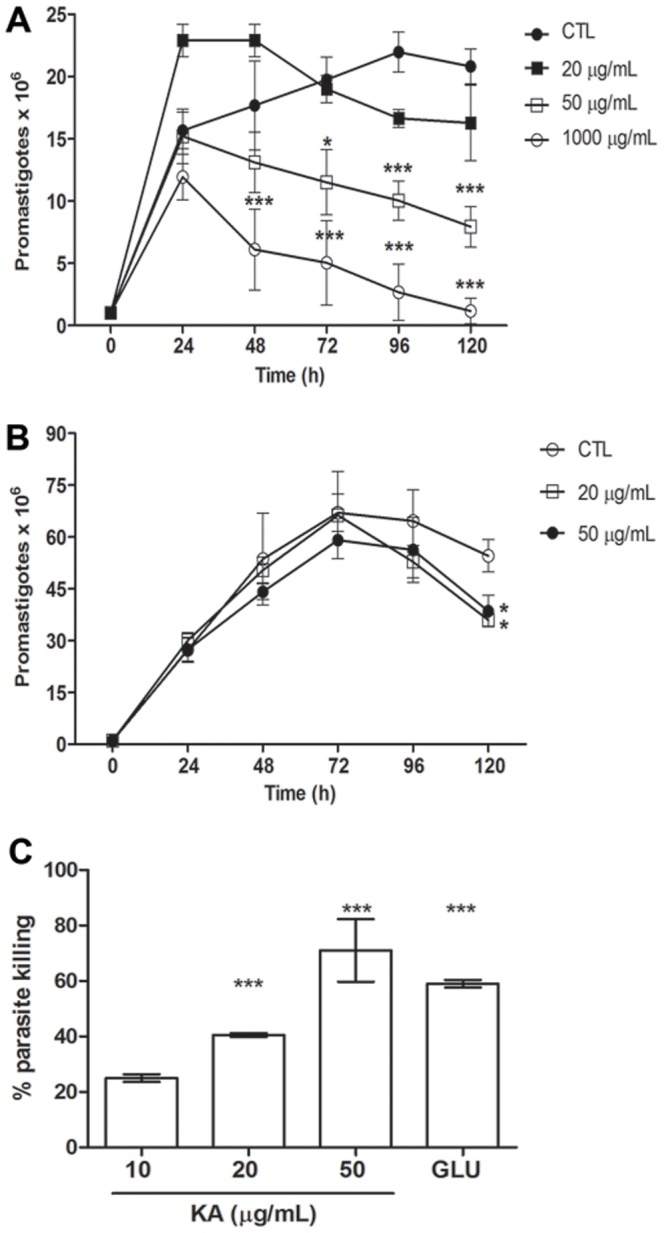
KA activity against *L. (L.) amazonensis in vitro*. (**A**) Growth curve of *L. (L.) amazonensis* promastigotes treated with different KA concentrations (B) Growth curve of *L. (L.) amazonensis* promastigotes treated with glucantime® (GLU). Results are from three experiments performed in triplicate. *p<0.05; ***p<0.001; compared with control (CTL). (**C**) Effect of KA on intracellular amastigote survival of *L. (L.) amazonensis*. Macrophages infected and treated with KA and GLU (50 μg/mL) (***p<0.001 compared with CTL).

The activity on *L. amazonensis* promastigotes forms was monitored for 120 hours. KA promoted a dose-dependent reduction of 62% in parasite proliferation, when treated with 50 μg/mL for 120 h (IC_50_ 34 μg/mL - Figure 1A). KA was 3-fold more effective than glucantime® reference drug (IC_50_ 122.4 μg/mL-Figure 1B), usually used for cutaneous leishmaniasis treatment in Amazon region. Leishmanicidal activity on intracellular parasites was evaluated after 72 h of treatment with KA. A growth inhibition of 79% (IC_50_ 27.9 μg/mL) after 72 h of treatment (Figure 1C). Glucantime® promoted a reduction of 59% after 72 h of treatment with 50 μg/mL (IC_50_ 77.4 μg/mL). MTT assay showed no cytotoxic effect on macrophage treated at 20–1000 μg/ml of KA when compared with control (Supplementary Figure S1).

### KA promotes ultrastructural alterations in amastigote forms of *L. amazonensis*

**Figure 2:**
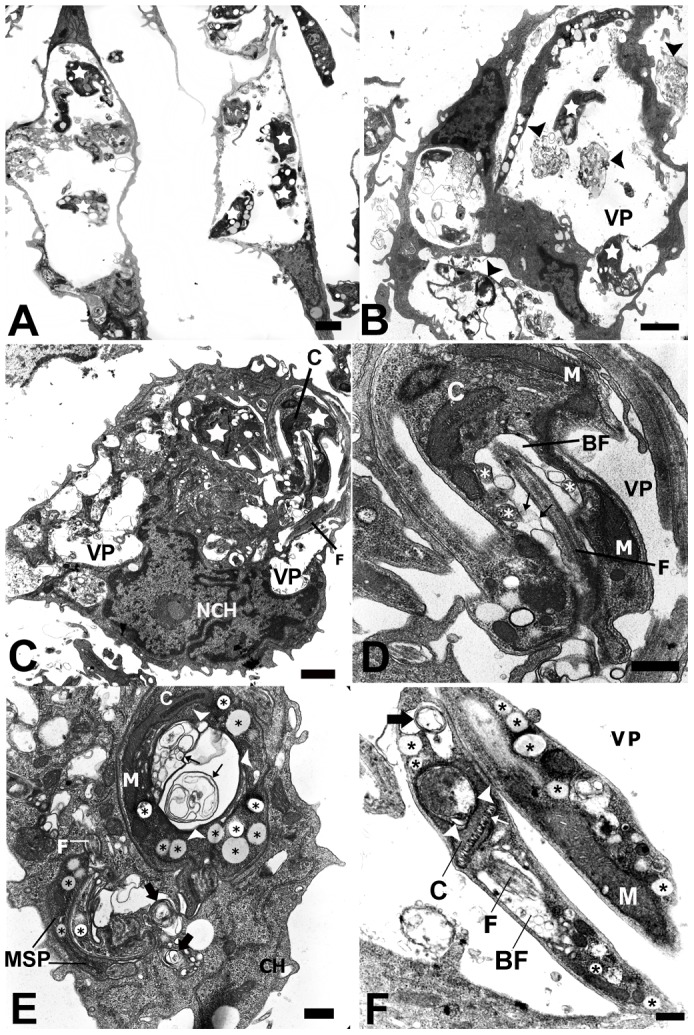
Ultrastructural effects of KA on intracellular amastigotes of *Leishmania (L.) amazonensis*. (**A**) General view of untreated infected macrophages showing a typical morphology. Note parasites (**stars**) within the parasitophorous vacuoles. (**B**) General view of infected macrophages treated for 1 h and cultivated for 24 h, showing large parasitophorous vacuole. Note reduced number of amastigotes, parasite and flagellar fragments (**arrowheads**). (**C**) Infected and treated macrophages presented vacuoles with damaged parasites (**stars**) or without amastigote forms. (**D**) Higher magnification of (**C**); parasites inside PV with alterations in the flagellar membrane (**thin arrows**) and vesicles inside the flagellar pocket (**asterisks**). (**E**) Intracellular amastigotes with membrane profiles in the flagellar pocket (**thin arrows**) and in the parasite cytoplasm (**arrows**); intense formation of lipid-like bodies (**asterisks**) in the cytoplasm of amastigotes forms. (**F**) Intracellular parasites presented concentric membrane (**arrow**), kinetoplast swelling (**arrow heads**) and lipid-like bodies (**asterisks**). *N*, nucleus; *FP*, flagellar pocket; *K*, kinetoplast; *F*, flagellum; *M*, mitochondria; *PV* parasitophorous vacuole. Bars: (**A–C**) 5 μm; (**D–F**) 2 μm.

**Figure 3:**
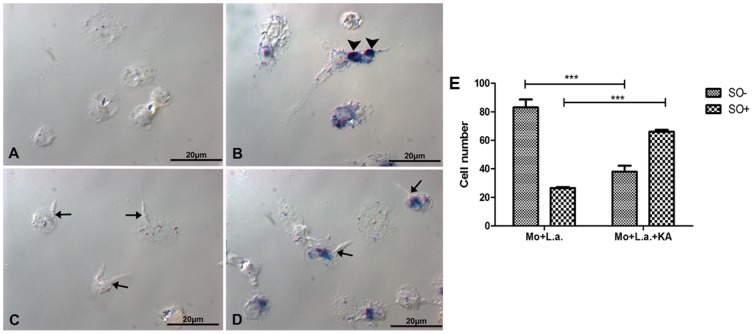
Superoxide radicals (O_2_^−^) detection in the infected macrophages treated with 50 μg/mL KA for 1 h. (**A**) Macrophages without infection and untreated. (**B**) Positive control, interaction with Zymozan (**arrows**). (**C**) Macrophage infected with *L. (L.) amazonensis* (**arrows**) showed superoxide production inhibition. (**D**) Macrophages infected with *L. amazonensis* and treated with KA reverted the inhibitory effect. Bars: 10 μm. (**E**) Number of infected macrophages that presented formazan deposits. MO+L.a.: macrophages infected with *L. amazonensis*; MO+L.a.+KA: macrophages infected with *L. amazonensis* and treated with 50 μg/mL KA; O_2_^+^: infected macrophages with formazan deposits; O_2_: infected macrophages without formazan deposites. (***p<0.001).

TEM was used to analyze alterations in the parasite organelles and as tool to elucidate the mechanisms of drug action. Infected cells showed that KA induced a decreased amastigote number (Figure 2B–C), with a large amount of membranous structures inside the parasitophorous vacuoles (Figure 3B-arrowheads). Furthermore, different and significant morphological alterations, such as some vesicles bodies into the flagellar pocket (Figure 2D), intense intracellular vacuolization (Figure 2F-asterisks), presence of many lipid-like bodies (Figure 2E-asterisks), and swelling of the mitochondrion (Figure 2F-arrowheads) were seen in the intracellular parasites.

### Infected macrophage treated with KA produced superoxide anions (O_2_^−^), but not nitric oxide

For superoxide detection, infected and treated macrophages were analyzed with a cytochemical assay using NBT. *Leishmania*-infected macrophages treated for 1 h with 50 μg/ml showed formazan deposits distributed in the cellular cytoplasm of infected cells, showing intense superoxide production (Figure 3D), in comparison with the infected-cells without KA-treatment (Figure 3C). The presence of formazan deposits enhanced (63%) when infected macrophage were treated 50 μg/ml KA as compared with infected macrophages without treatment. On the other hand, nitric oxide (NO) production was not observed in infected macrophages that were treated with 50 μg/ml of KA (data not shown).

### KA- topical formulation (ointment) promoted amastigote destruction *in vivo*

**Figure 4:**
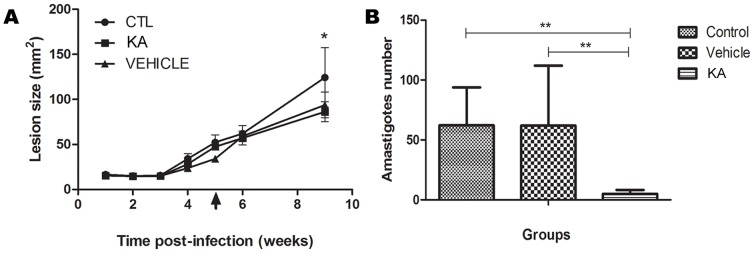
Effect of KA topical formulation on experimental infection with *L. amazonensis*. (**A**) Development of lesions in *L. (L.) amazonensis*-infected animals treated with KA. The treatment started after 5 weeks post-infection and continued for 4 weeks. Data represent the average measurements of 5 animals for each group. (**B**) Parasite load in the lesion sites. Amastigotes were quantified after interruption of treatment and the mean number of parasites evaluated in 10 fields reported; (**p<0.001). Results are expressed as the mean number of cells evaluated in 10 fields; (*p<0.05; ***p<0.0001).

After the end of the treatment, tissue samples were collected. Lesion size was measure weekly during the treatment period. Surprisingly, there was a low, but significant, reduction in lesion size in the end of 4 weeks of treatment (Figure 4A), associated with a reduced number of amastigote forms (Figure 4B). Topical treatment with KA ointment decreased the parasite number at the lesion site by 92.1%. Interestingly, the vehicle group presented a discrete reduction in lesion size, but no decrease in amastigote number (Figure 4B). In control group, lesion size enhanced and a large number of amastigotes were detected (Figure 4A and 4B).

### KA-topical formulation promoted collagen production

**Figure 5:**
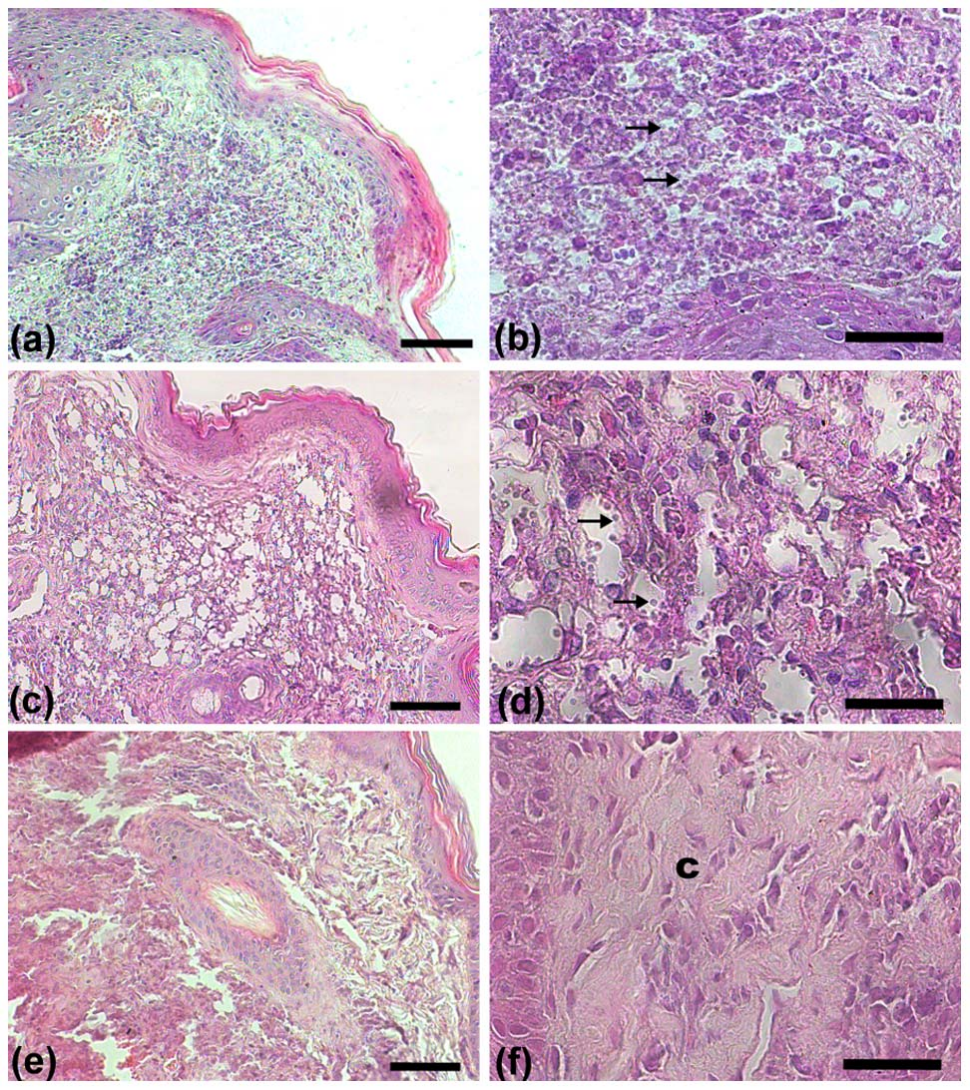
Skin lesion section of infected animals, untreated and treated with KA topical formulation for one month. Histopathologycal analysis of lesion site of untreated animal (**A–B**) showed tissue damage (**A**) and numerous amastigote forms (**arrows**) dispersed for all tissue (**B**); Vehicle group (**C–D**) showed an intense vacuolization for all tissue (**C**) and a higher number of amastigotes (**arrows**) inside the parasitophorous vacuoles (**D**); KA-treated animals (**E–F**) presented reduced number of vacuoles in the macrophages and reduced number of amastigotes (**E–F**); H&E stain. Bars: (**A, C, E**) 20 μm; (**B, D, F**) 10 μm.

**Figure 6:**
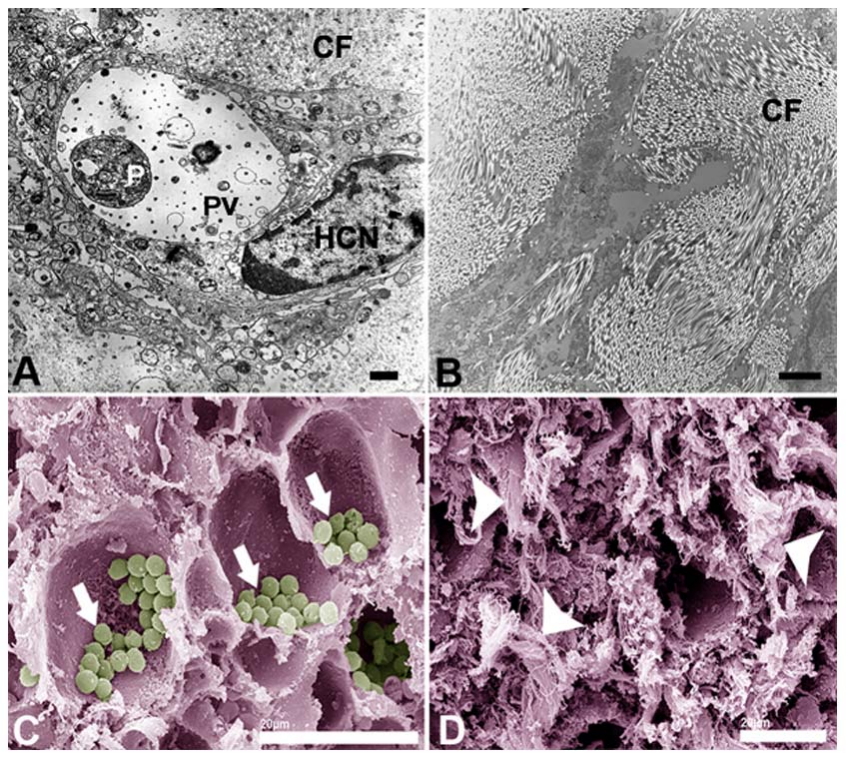
Ultrastructural analysis of skin lesions from infected animals untreated and treated with KA-topical formulation. Transmission electron microscopy of control (**A**) and KA-treated animals (**B**). Note the presence of amastigotes in the parasitophorous vacuoles and few collagen fibers in the control group and absence of intracellular amastigotes with organized fibers in the treated group. Scanning electron microscopy of untreated lesion showed the presence of a large number of amastigotes (**arrows**) inside the parasitophorous vacuoles (**C**) and lesion from treated animals showed an intense production of fibrous material suggestive of collagen fibers (**D-arrowheads**). *P*, parasite; *HCN*, host cell nuclei; *PV*, parasitophorous vacuole. Bars: (**A–B**) 3 μm, (**C–D**) 20 μm.

In control group was observed a large number of amastigotes dispersed for all tissue (Figure 5A–B). Vehicle group presented many amastigotes forms inside of host cell vacuoles (Figure 5C–D). On the other hand, KA-treated group presented lower number of amastigotes and collagen fibers in the infection site, confirmed by picrosirius red stain (Figure 5E–F). These fibers demonstrated an organized and parallel distribution in treated animals and seem to fill the spaces between host cell vacuoles (See supplementary movies S1 and S2). Ultrastructural analysis by TEM and SEM of treated group were performed. Analysis by TEM of treated group showed a predominant parallel distribution of collagen fibers (Figure 6B) when compared to control that presented amastigotes forms inside parasitophorous vacuoles (Figure 6A). As well as, analysis by SEM of control group demonstrated higher number of amastigotes inside vacuoles (Figure 6C-arrows). In contrast, KA-treated group presented empty vacuoles and a large number of collagen fibers in almost all recovered tissue (Figure 6D-arrowheads).

## Discussion

It is well known that chemotherapy is the only effective treatment for Leishmaniasis infections, however, the antileishmanial drugs available are, in general, toxic expensive and require long-term treatment; furthermore, most of them can only be given to patients parenterally. These side effects and disadvantages demonstrate the necessity to identify new, effective and safe compounds for the treatment of this disease [4], [26].

In the present study, we showed that KA acted as an antileishmanial agent without affecting the host cell. KA treatment decreased the growth of *L. amazonensis* promastigotes by 62% at 50 μg/mL (IC_50_: 34 μg/mL). Moreover, KA was more effective against intracellular amastigotes, with a growth inhibition of 79% for 50 μg/mL of KA after 72 h of treatment (IC_50_: 27.84 μg/mL).

Recently, we reported that KA could modulate macrophage activation through cytoskeleton rearrangement, increase cell surface exposure, and enhance the phagocytic process and superoxide anions (O_2_^−^) production [18]. The antiamastigote effect observed in the present report showed that KA promotes a stimulatory effect in macrophages, killing the parasites. Macrophages infected and treated for just 1 h were able to produce O_2_^−^, even in the presence of *L. amazonensis*. It is known that *L. amazonensis* can evade the macrophage microbicidal action, inhibiting reactive oxygen species (ROS) and NO production [27], [28] and KA was able to reverse this inhibitory process. The mechanism by which KA exerts its antileishmanial effects *in vitro* seems to involve the activation of macrophages and amastigotes killing by O_2_^-^production rather than the action of NO. In agreement with our results, Valadares *et al*. (2011) [29] demonstrated that the *Agaricus blazei* Murill mushroom presents effects against intracellular amastigotes with a reduction of 84.4% by a mechanisms that are NO-independent.

We also analyzed the effects of KA on parasite ultrastructure. Identification of morphological changes can help elucidate the mechanisms of drug action [30]. In intracellular amastigotes, significant morphological alterations were observed, such as intense intracellular vacuolization, disruptions in flagellar membrane and the presence of many vesicles bodies in the flagellar pocket. Different natural compounds with anti-leishmanial activity induce alterations in this particular region of the parasite, suggesting alterations in the endocytic/exocytic pathway [6], [31]. Other characteristic features seen in intracellular amastigotes treated with KA, for 72 h was the presence of myelin-like figures and the over-accumulation of lipid-like bodies in the cytoplasm. Studies have shown that myelin-like figures are correlated to the autophagic process during drug action [32]. This process is associated with the cell response to starvation or stresses [33] and is dependent on ROS production [34]. With respect to lipid body accumulation in the parasites, close association has been reported with increased lipid body production, ROS production and autophagy [34]. KA seems to induce lipid droplet formation though ROS activity in the host cell and consequently lead to autophagic cell death. However, further studies are necessary to clarify these questions.

Due to the leishmanicidal effect of KA upon intracellular amastigotes *in vitro*, we examined the effect *in vivo* with a KA-topical formulation in the animal model of cutaneous leishmaniasis. Several new topical treatments have been made available to treat this disease, but well-known drugs, such as imiquimod and paromomycin, are the only ones that have reached the clinical trial testing phase as a topical formulation (ointment, cream, gel and solution) [35], [36]. Nevertheless, the results obtained are still a controversial interpretation of results, complicated by nonstandard definitions of the disease and its cure.

In this study, infected animals treated with KA-topical formulation promoted an initiation of healing process, due to the production of numerous collagen fibers at the infection site, as well as a decrease in parasite burden, after the end of treatment. Wound healing is a process characterized by inflammation, cell proliferation and tissue remodeling where neutrophils, macrophages and lymphocytes arrive first, followed by fibroblasts [37]. *Leishmania* produces proteases that are capable of destroying matrix proteins, and disrupting dermal barriers to propagate infection [38]. However, our present data also show that KA promoted the production of a large number of type I collagen fibers over the type III fibers in the infected animals. The presence of type III fibers is suggested to be related to the success of parasitism, since these fibers provide support to inflammatory cells, such as vacuolated and parasitized histiocytes [38]. It has been reported that, in murine models, the presence of collagen fibers is prominent in areas where new epithelium is produced, and where parasites have been eliminated and the inflammatory process controlled [39]. Furthermore, it has been demonstrated that KA enhances the wound-healing process following topical application [40].

Interestingly, our data show a discrete decrease in the lesion size, in association with the presence of numerous, aligned and parallel collagen fibers, and the absence or a low-level of parasites. Thus, not only could lesion size determine the long-term outcome of tissue damage and the repair process, but this could also influence the killing of parasites and parasite environment modification [41], [42]. Besides, the small decrease in lesion size in KA-treated animals compared to control animals can be explained by the fact that the intensive collagen fibers production can be related to the beginning of the healing process, where there has not been enough time to occurred tissue remodeling, the final stage of wound healing [43].

Another interesting observation is that animals that received only the treatment with vehicle (triacylglycerols obtained from the fruit seed of *Theobroma grandiflorum*) showed a similar decrease in lesion size when compared to in KA treated animals. However, when histological analysis of lesion was performed, the vehicle group showed a lot of amastigotes when compared to the treated group. The vehicle from *Theobroma grandiflorum* seeds seems to help the healing process, but not the parasite death; however, further studies should be conducted to determine its healing potential.

The present findings indicate that KA seems to act indirectly against the parasite and promotes, initially, the activation of macrophages, leading to a O_2_^-^production that is able to kill the *L. amazonensis* parasite and help to control the infection. Thus, KA could be useful for the selective treatment of cutaneous leishmaniasis and may hold great potential as an anti-leishmanial agent. To our knowledge, this report demonstrates, for the first time, the action of KA on *Leishmania amazonensis in vitro* and *in vivo*. This study forms part of a continual search for new bioactive products, obtained from biotechnological processes that can act effectively against neglected diseases, such as leishmaniasis.

## Supporting Information

Figure S1 **MTT assay to determine macrophage viability.** Formazan result solution was read at plate reader at 550 nm and absorbance represents cells viability. No differences were found at 20–1000 μg/ml of KA when compared with the control. OD: optical density; CTL: control.
(TIF)

Movie S1 **Z-stack confocal images of control group stained with picrosirius red for collagen detection.** A total of 13 images were collected and the distance between planes is 0.98 μm. (630x).
(MPG)

Movie S2 **Z-stack confocal images KA-treated group stained with picrosirius red for collagen detection.** A total of 8 images were collected and the distance between planes is 0.98 μm. (630x).
(MPG)

## References

[1] Lainson R (2010) The Neotropical Leishmania species: a brief historical review of their discovery, ecology and taxonomy. Rev Pan-Amaz Saude 1: 13–32.

[2] Silveira FT, Lainson R, Corbett CEP (2004) Clinical and immunopathological spectrum of American cutaneous leishmaniasis with special reference to the disease in Amazonian Brazil: a review. Mem Inst Oswaldo Cruz 99: 239–251. 15273794 10.1590/s0074-02762004000300001

[3] Silveira FT (2009) Diffuse cutaneous leishmaniasis (DCL) in the Amazon region, Brazil: clinical and epidemiological aspects. Gaz Med Bahia 79: 25–29.

[4] Croft SL, Sundar S, Fairlamb AH (2006) Drug resistance in Leishmaniasis. Clin Microbiol Rev 19: 111–126. 16418526 10.1128/CMR.19.1.111-126.2006 PMC1360270

[5] Polonio T, Efferth T (2008) Leishmaniasis: Drug resistance and natural products (Review). Int J Mol Med: 277–286. 18698485

[6] Guimarães LRC, Rodrigues APD, Marinho PSB, Muller AH, Guilhon GM, et al (2010) Activity of the julocrotine, a glutarimide alkaloid from *Croton pullei* var. *glabrior*, on *Leishmania (L.) amazonensis* . Parasitol Res 107: 1075–1081. 20661748 10.1007/s00436-010-1973-0

[7] Burdock GA, Soni MG, Carabin IG (2001) Evaluation of health aspects of kojic acid in food. Regul Toxicol Pharmacol 33: 80–101. 11259181 10.1006/rtph.2000.1442

[8] Blumenthal CZ (2004) Production of toxic metabolites in *Aspergillus niger*, *Aspergillus oryzae*, and *Trichoderma reesei*: justification of mycotoxin testing in food grade enzyme preparations derived from the three fungi. Regul Toxicol Pharmacol 39: 214–228. 15041150 10.1016/j.yrtph.2003.09.002

[9] Bentley R (2006) From miso, saké and shoyu to cosmetics: a century of science for kojic acid. Nat Prod Rep 23: 1046–1062. 17119644 10.1039/b603758p

[10] Lim JT (1999) Treatment of melasma using kojic acid in a gel containing hydroquinone and glycolic acid. Dermatol surg 25: 282–284. 10417583 10.1046/j.1524-4725.1999.08236.x

[11] Nohynek GJ, Kirkland D, Marzin D, Toutain H, Leclerc-Ribaud C, et al (2004) An assessment of the genotoxicity and human health risk of topical use of kojic acid [5-hydroxy-2-(hydroxymethyl)-4H-pyran-4-one]. Food Chem Toxicol 42: 93–105. 14630133 10.1016/j.fct.2003.08.008

[12] Lin CH, Wu HL, Huang YL (2007) Combining high-performance liquid chromatography with on-line microdialysis sampling for the simultaneous determination of ascorbyl glucoside, kojic acid, and niacinamide in bleaching cosmetics. Anal Chim Acta 581: 102–107. 17386432 10.1016/j.aca.2006.08.002

[13] Ha YM, Chung SW, Song S, Lee H, Suh H, et al (2007) 4-(6-Hydroxy-2-naphthyl)-1,3-bezendiol: a potent, new tyrosinase inhibitor. Biol Pharm Bull 30: 1711–1715. 17827726 10.1248/bpb.30.1711

[14] Gomes AJ, Lunardi CN, Gonzalez S, Tedesco AC (2001) The antioxidant action of *Polypodium leucotomos* extract and kojic acid: reactions with reactive oxygen species. Braz J Med Biol Res 34: 1487–1494. 11668361 10.1590/s0100-879x2001001100018

[15] Tamura T, Mitsumori K, Totsuka Y, Wakabayashi K, Kido R, et al (2006) Absence of in vivo genotoxic potential and tumor initiation activity of kojic acid in the rat thyroid. Toxicology 222: 213–224. 16603304 10.1016/j.tox.2006.02.023

[16] Moto M, Mori T, Okamura M, Kashida Y, Mitsumori K (2006) Absence of liver tumor-initiating activity of kojic acid in mice. Arch Toxicol 80: 299–304. 16231124 10.1007/s00204-005-0034-4

[17] Emami S, Hosseinimehr SJ, Taghdisi SM, Akhlaghpoor S (2007) Kojic acid and its manganese and zinc complexes as potential radioprotective agents. Bioorg Med Chem Lett 17: 45–48. 17049858 10.1016/j.bmcl.2006.09.097

[18] Rodrigues APD, Carvalho ASC, Santos AS, Alves CN, Do Nascimento JLM, et al (2011) Kojic acid, a secondary metabolite from Aspergillus sp., acts as an inducer of macrophage activation. Cell Biol Int 35: 335–343. 21044044 10.1042/CBI20100083

[19] Santos AS, Silva EO, Nascimento JLM, Alves CN, Carvalho ASC, et al.. (2010) Use of 5-hydroxy-2-hydroxymethyl-γ-pyrone as a macrophage activation agent to combat cutaneous leishmaniasis. European patent WO2010017613.

[20] Santos AS, Silva EO, Nascimento JLM, Alves CN, Carvalho ASC, et al.. (2011) Uso do 5-hidroxi-2-hidroximetil-γ-pirona como agente de ativação do macrófago no combate da Leishmaniose Cutânea. Brazilian patent PI0817954-9.

[21] Santos AS, Silva EO, Nascimento JLM, Alves CN, Carvalho ASC, et al.. (2011) Use of 5-hydroxy-2-hydroxymethyl-γ-pyrone as a macrophage activation agent to combat cutaneous leishmaniasis. U.S. patent US20110178169A1.

[22] Fitzpatrick JM, Hirai Y, Hirai H, Hoffmann KF (2007) Schistosome egg production is dependent upon the activities of two developmentally regulated tyrosinases. FASEB J 21: 823–835. 17167065 10.1096/fj.06-7314com

[23] Ferreira NR, Sarquis MIM, Alves CN, Santos AS (2010) Biotransformation of sucrose into 5-hydroxy-2-hydroxymethyl-γ-pirone by Aspergillus flavus. Annals of the Brazilian Academy of Sciences 82: 569–576. 10.1590/s0001-37652010000300004 21562685

[24] Rocha-Vieira E, Ferreira E, Vianna P, et al (2003) Histopathological outcome of Leishmania major-infected BALB/c mice is improved by oral treatment with N-acetyl-l-cysteine. Immunology 108: 401–408. 12603607 10.1046/j.1365-2567.2003.01582.x PMC1782893

[25] Haggis GH, Phipps-Todd B (1977) Freeze-fracture for scanning electron microscopy. J Microsc 111: 193–201. 599555 10.1111/j.1365-2818.1977.tb00059.x

[26] Croft SL, Seifert K, Yardley V (2006) Current scenario of drug development for leishmaniasis. Indian J Med Res 123: 399–410. 16778319

[27] Olivier M, Gregory DJ, Forget G (2005) Subversion mechanisms by which leishmania parasites can escape the host immune response: a signaling point of view. Clin Microbiol Rev 18: 293–305. 15831826 10.1128/CMR.18.2.293-305.2005 PMC1082797

[28] Mukbel RM, Patten C, Gibson K, Ghosh M, Petersen C, et al (2007) Macrophage killing of *Leishmania amazonensis* amastigotes requires both nitric oxide and superoxide. The Am J Trop Med Hyg 76: 669–675. 17426168

[29] Valadares DG, Duarte MC, Oliveira JS, Chávez-Fumagalli MA, Martins VT, et al (2011) Leishmanicidal activity of the *Agaricus blazei* Murill in different *Leishmania* species. Parasitol Int 60: 357–363 doi:10.1016/j.parint.2011.06.001 21723957 10.1016/j.parint.2011.06.001

[30] Adade CM, Souto-Padrón T (2010) Contributions of ultrastructural studies to the cell biology of trypanosmatids: targets for anti-parasitic drugs. Open Parasitol J 4: 178–187.

[31] Vendrametto MC, Santos AO, Nakamura CV, Dias Filho BP, Cortez DA, et al (2010) Evaluation of antileishmanial activity of eupomatenoid-5, a compound isolated from leaves of *Piper regnellii* var. *pallescens* . Parasitol Int 59: 154–158. 20064628 10.1016/j.parint.2009.12.009

[32] Rodrigues JCF, Rodriguez C, Urbina JA (2002) Ultrastructural and biochemical alterations induced by promastigote and amastigote forms of *Leishmania amazonensis* . Curr Pharm Des 46: 487–499. 10.1128/AAC.46.2.487-499.2002 PMC127026 11796362

[33] Kondo Y, Kanzawa T, Sawaya R, Kondo S (2005) The role of autophagy in cancer development and response to therapy. Nat Rev Cancer 5: 726–734. 16148885 10.1038/nrc1692

[34] Djavaheri-Mergny M, Amelotti M, Mathieu J, Besançon F, Bauvy C, et al (2006) NF-KB activation represses tumor necrosis factor-alpha-induced autophagy. J Biol Chem 281: 30373–30382. 16857678 10.1074/jbc.M602097200

[35] Alavi-Naini R, Fazaeli A, O'Dempsey T (2012) Topical treatment modalities for old world cutaneous leishmaniasis: a review. Prague Med Rep 113: 105–118. 10.14712/23362936.2015.26 22691282

[36] Croft SL, Olliaro P (2011) Leishmaniasis chemotherapy—challenges and opportunities. Clin Microbiol Infect 17: 1478–1483. 21933306 10.1111/j.1469-0691.2011.03630.x

[37] Miller M, Nanchahal J (2005) Advances in the modulation of cutaneous wound healing and scarring. BioDrugs 19: 363–381. 16392889 10.2165/00063030-200519060-00004

[38] Abreu-Silva AL, Calabrese KS, Mortara RA, Tedesco RC, Cardoso FO, et al (2004) Extracellular matrix alterations in experimental murine *Leishmania (L.) amazonensis* infection. Parasitology 128: 385–390. 15151143 10.1017/s0031182003004621

[39] Baldwin T, Sakthianandeswaren A, Curtis JM, Kumar B, Smyth GK, et al (2007) Wound healing response is a major contributor to the severity of cutaneous leishmaniasis in the ear model of infection. Parasite Immunol 29: 501–513. 17883453 10.1111/j.1365-3024.2007.00969.x

[40] Mohammadpour M, Behjati M, Sadeghi A (2012) Wound healing by topical application of antioxidant iron chelators: kojic acid and deferiprone. Int Wound J doi:10.1111/j.1742–481X.2012.00971.x. 10.1111/j.1742-481X.2012.00971.x PMC7950824 22621771

[41] Sakthianandeswaren A, Elso CM, Simpson K, Curtis JM, Kumar B, et al (2005) The wound repair response controls outcome to cutaneous leishmaniasis. Proc Natl Acad Sci U S A 102: 15551–15556. 16223880 10.1073/pnas.0505630102 PMC1266107

[42] Lecoeur H, Buffet P, Morizot G, Goyard S, Guigon G, et al (2007) Optimization of topical therapy for *Leishmania major* localized cutaneous leishmaniasis using a reliable C57BL/6 model. PLoS Negl Trop Dis 1: e34. 18060082 10.1371/journal.pntd.0000034 PMC2100369

[43] Nurden A T (2011) Platelets, inflammation and tissue regeneration. Thromb Haemost 105(suppl (6)) S13–33. 21479340 10.1160/THS10-11-0720

